# Case Report: A Nasopharyngeal Cancer Patient Got COVID-19 During Radiochemotherapy in Wuhan

**DOI:** 10.3389/fonc.2020.01755

**Published:** 2020-10-27

**Authors:** Weiming Qiu, Fengtao Yi, Xingfeng Ren, Zhaoxia Li, Deqiang Wang, Ning Wang

**Affiliations:** ^1^Department of Burn and Plastic Surgery, General Hospital of Central Theater Command of Chinese People's Liberation Army, Wuhan, China; ^2^Department of Infectious Diseases, General Hospital of Central Theater Command of Chinese People's Liberation Army, Wuhan, China; ^3^Department of Radiotherapy, General Hospital of Central Theater Command of Chinese People's Liberation Army, Wuhan, China; ^4^Department of Urology, General Hospital of Central Theater Command of Chinese People's Liberation Army, Wuhan, China; ^5^Rocket Force Characteristic Medical Center of Chinese People's Liberation Army, Beijing, China; ^6^Department of Medical Oncology, Affiliated Hospital of Jiangsu University, Zhenjiang, China

**Keywords:** COVID-19, SARS-CoV-2, nasopharyngeal cancer, radiochemotherapy, lymphocyte, recovery

## Abstract

The coronavirus disease (COVID-19) infection, caused by the novel severe acute respiratory syndrome coronavirus 2 (SARS-CoV-2), has spread worldwide. Reports of COVID-19 among cancer patients are limited and most studies focus mainly on its epidemiological and clinical features. In this study, we report the case of a nasopharyngeal cancer patient from Wuhan who contracted COVID-19 during radiochemotherapy and has since recovered from the infection. We hope that this report will provide valuable insight into the treatment of SARS-CoV-2-infected cancer patients through an account of our experience.

## Introduction

Since December 2019, coronavirus disease (COVID-19), caused by a novel coronavirus named severe acute respiratory syndrome coronavirus 2 (SARS-CoV-2), emerged in Wuhan, Hubei province, China, and spread worldwide. Due to the reduced accessibility of cancer care services caused by the COVID-19 pandemic, cancer patients have been suffering from dual anxiety due to SARS-CoV-2 infection and cancer progression. Reports of COVID-19 in cancer patients have been limited so far, and most studies have focused mainly on the epidemiological and clinical features of the infection (1–3). To the best of our knowledge, cases of cancer patients who contracted COVID-19 during chemotherapy or radiotherapy have not been reported in detail. Nasopharyngeal cancer patients deserve more attention since the nasopharyngeal cavity is a crucial pathway by which this respiratory virus invades the lungs and is the primary reservoir of SARS-CoV-2 (4). Due to the potential adverse effects of chemo/radiotherapy on immunity, there is growing fear of receiving treatment among cancer patients. Therefore, more accurate reports from the frontlines are valuable to policy makers and oncologists alike. Herein, we report the case of a nasopharyngeal cancer patient who contracted COVID-19 during radiochemotherapy and has since recovered from the infection. The case occurred in Wuhan, the original epicenter of the outbreak in China.

## Case Description

**Figure 1:**
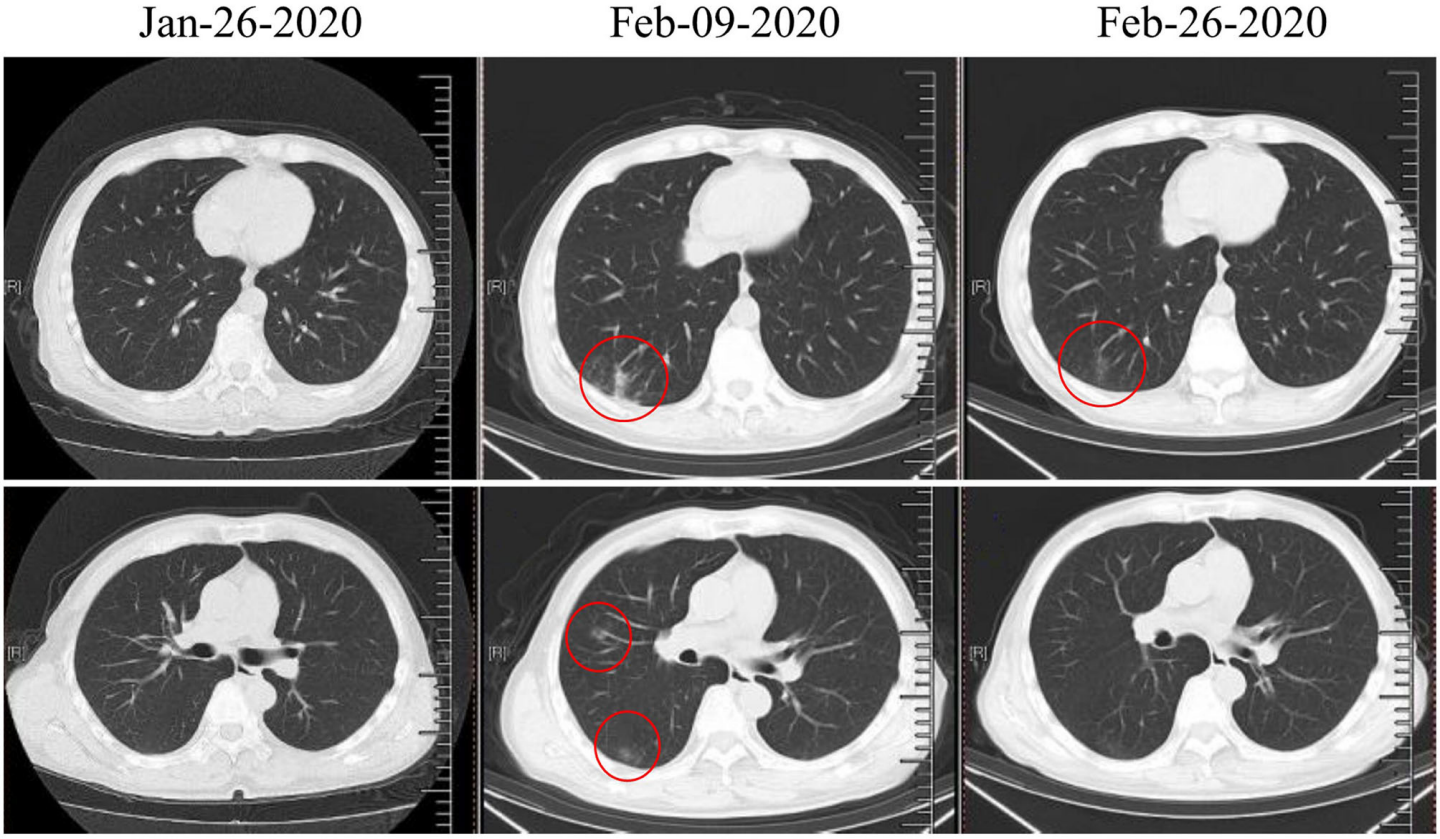
Representative computed tomographic images of three time points. January 26, 2020, day of first fever; February 9, 2020, day of second fever and diagnosis of coronavirus disease (COVID-19); February 26, 2020, 2 days before discharge. Areas of pneumonia are marked with red circles.

**Figure 2:**
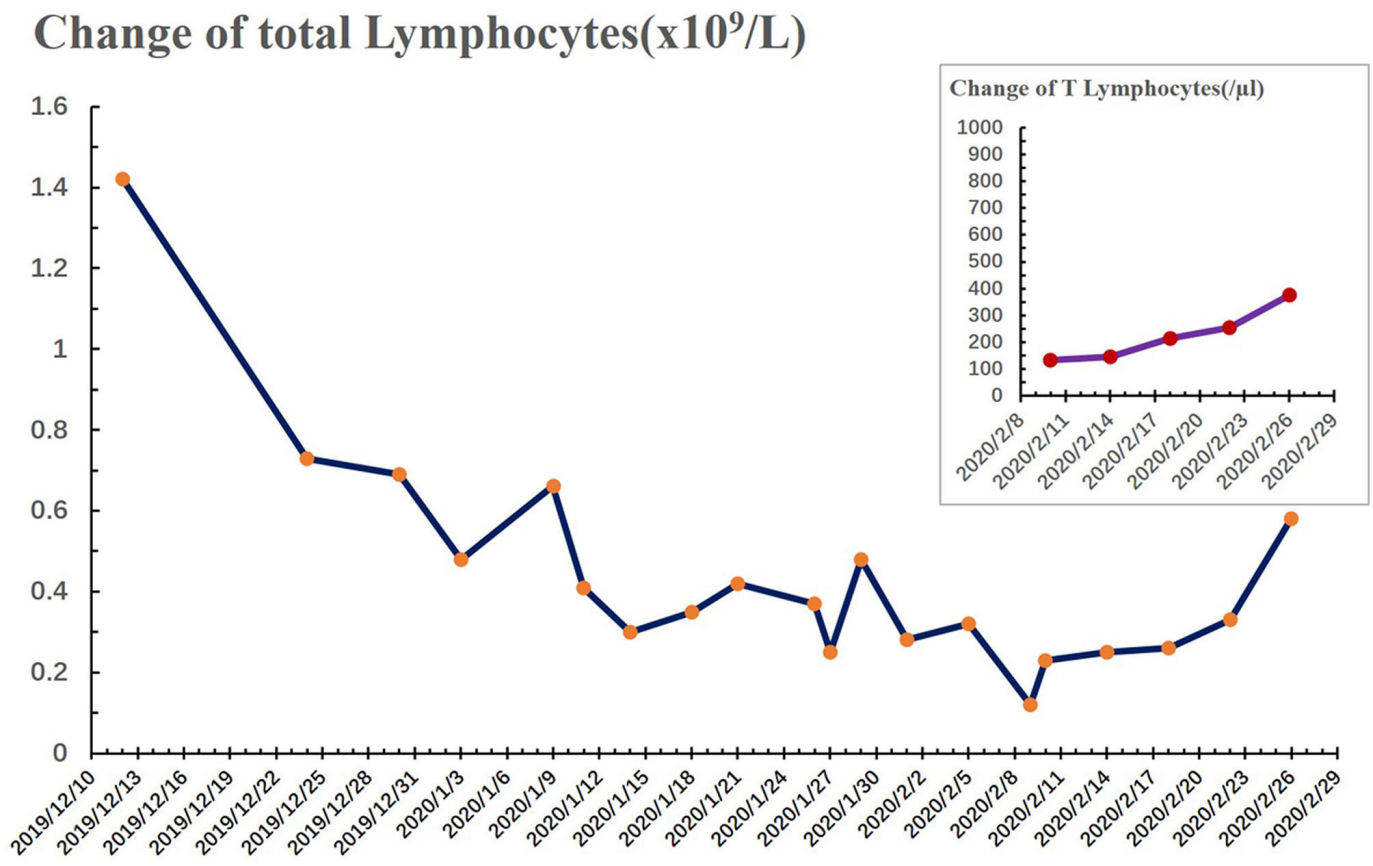
Change in total lymphocytes (dark blue) and T lymphocytes (purple) during hospitalization. The number of total lymphocytes reached the lowest at 0.12 × 10^9^ cells/L on February 9, 2020. The lower limit of the normal range of total lymphocytes and T lymphocytes is marked in the figure (dashed line).

On December 11, 2019, a 51-year-old male visited our outpatient clinic located in Wuhan, China complaining of “decreased hearing ability and bloody sucked-back snot.” He had undergone hypophysoma resection in 2003 and had been suffering from chronic kidney disease since 2010. Imaging and biopsies confirmed the diagnosis of nasopharyngeal non-keratinizing squamous cell carcinoma (cT3N1M0). Although routine examination after admission indicated anemia (Grade 1), hypoalbuminemia (Grade 1), and hypothyroidism, he was deemed eligible to receive radiochemotherapy (KPS = 80). As shown in Table 1, he received chemotherapy (lobaplatin 35 mg/m^2^, d1, d22) on December 19, 2019 and January 9, 2020, and radiotherapy (PTV70 69.96Gy/33F) from December 23, 2019 to February 7, 2020. During the treatment, adverse effects, including oral mucositis (Grade 1–2), nausea (Grade 1), anorexia (Grade 1–3), fatigue (Grade 1), hyponatremia (Grade 1–4), and neutropenia (Grade 2) occurred. In addition, there was persistence and aggravation of his pre-existing anemia (Grade 1–2). The patient once intended to quit due to the pain from mucositis (Mucosal hyperemia and ulcer found by physical examination) and weight loss (lost 7.5 kilograms when chemotherapy ended) caused by anorexia. However, he finally completed all planned therapies with psychological support from medical care providers, and all of the adverse effects improved partially or completely after symptomatic treatment. On January 26, 2020, the patient suddenly developed a high fever (>39°C, axillary temperature) accompanied by fatigue and mild dysphoria but reported no cough, shortness of breath, or loss of smell or taste. Meanwhile, no abnormal signs were found during physical examination and no sign of pneumonia was found on chest computerized tomography (CT) (Figure 1). The fever was rapidly relieved by symptomatic treatment. After completion of radiochemotherapy, he developed a fever again (38°C, axillary temperature) on February 9 accompanied by nasal congestion, dry throat, and fatigue. Physical examination found no obvious positive sign, but the chest CT showed multiple flaky ground-glass opacities and patches in the right lung, suggestive of viral pneumonia (Figure 1). Since the COVID-19 epidemic was ongoing in Wuhan at the time, he was promptly transferred to the infectious disease ward, the SARS-CoV-2 test was shortly performed on a pharyngeal swab sample, and the result came out positive the next day. Additional tests ruled out Influenza A, Influenza B, and other infections caused by common respiratory tract pathogens. He underwent 2 weeks of comprehensive treatment (Table 1) including oseltamivir (75 mg, twice a day), ribavirin (0.5 g, once a day), Lianhua-Qingwen capsule (originating from traditional Chinese herbs, 1.05 g, three times a day), thymopentin (10 mg, once a day), immune globulin Ph4 (10 g, once a day), recombinant human interferon-α1B (300 million units, once a day), and supportive treatments such as intravenous nutrition therapy. No obvious side effects were observed during the treatment of COVID-19 with the above drugs. In late February, his symptoms were relieved in part or totally (Table 1), and the chest CT taken on February 26, 2020 revealed a marked resolution of pneumonia (Figure 1). Multiple SARS-CoV-2 tests were carried out until two successive negative results were obtained. During the hospitalization, we found the lymphocyte counts were highly correlated with the change of disease (Figure 2). It gradually decreased after radiochemotherapy and reached its lowest count (0.12 × 10^9^/L) on February 9, 2020, the same day as the diagnosis of viral pneumonia. Since then, a steady rebound was observed on both counts of T lymphocytes and total lymphocytes. On February 28, the patient left the hospital and went to a community-designated accommodation for medical observation under quarantine for 14 days. He received SARS-CoV-2 tests four times in a month after discharge (twice during the quarantine and twice later in the community) and all results were negative. He was examined at another oncology center on May 12, 2020 and the evaluation of the response to radiochemotherapy was “clinical complete response.” In the most recent telephone follow-up on June 9, 2020, he was in good condition and had gained 6 kilograms since January 7, 2020, the end of radiochemotherapy, and there has been no relapse of the previously reported symptoms after discharge.

**Table 1:** Timeline from first chemotherapy to discharge.

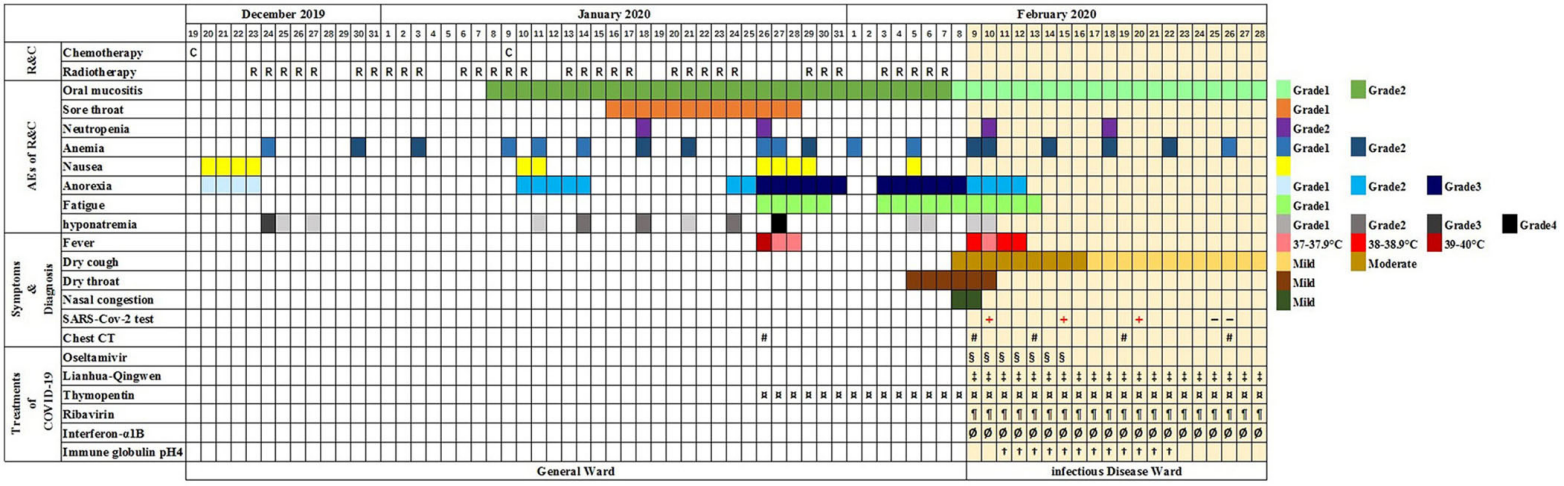

*The schedule of radiochemotherapy (R & C) and adverse events (AE), onset of symptoms, and diagnosis and treatment of coronavirus disease (COVID-19) are marked with colors and symbols. The planed radiotherapy on January 27, 2020 and January 28, 2020 were delayed due to the patient's fever. The severity of AE was graded according to the Common Terminology Criteria for Adverse Events (CTCAE) version 5*.

## Discussion

Cancer patients are predominantly elderly and immunosuppressed, and preliminary clinical data suggest that they may be more susceptible to SARS-CoV-2 infection (1, 2). Meanwhile, radiotherapy and chemotherapy could further damage the mucosa and immune system. Furthermore, the nasopharyngeal cavity is an important stop on the transmission pathway of the respiratory virus to the lungs. Studies have found that the nasopharyngeal cavity is the main reservoir of SARS-CoV-2 (4). All these factors together increase the risk of infection among nasopharyngeal cancer patients. In this case, typical symptoms of COVID-19 such as fever, dry cough, and fatigue developed, but not loss of smell or taste, a symptom recently reported in more than 60% of COVID-19 patients (5, 6). Lymphocytes, especially T-lymphocytes, have been described as the potential targets of SARS-CoV-2 (7). As the correlation showed both counts and the disease changing in this case, we thought that the rising of total lymphocyte and T-lymphocyte counts after treatment may facilitate his recovery. Their value of prediction on early diagnosis and the treatment's efficacy deserve further study. According to a recent study from Wuhan (3), this patient had two characteristics that put him at greater risk of developing severe events: being diagnosed with COVID-19 immediately after completion of radiochemotherapy and the appearance of ground-glass opacities and patchy consolidations on chest CT. Nevertheless, he recovered in a short time. However, other than some preliminary data that have not been peer-reviewed, there is still no persuasive clinical evidence on the efficacy of the drugs that we used to treat him for COVID-19. Therefore, effective approaches to treat COVID-19 supported by evidence and additional research on immunity are required. Along with the development of targeted therapy, orally-administered drugs play a vital role in the treatment of some cancers, e.g., EGFR-TKI for non-small cell lung cancer, which became a priority during the pandemic. Nevertheless, radiotherapy and chemotherapy are still the main approaches to treat nasopharyngeal cancer, which makes treatment planning for this type of cancer more challenging at present.

Based on a study reporting the detection of SARS-CoV-2 infection relative to symptom onset (8), we speculate that the infection happened during radiochemotherapy, although we could not identify the exact date. During hospitalization in the general and infectious diseases wards, no medical staff member who took care of this patient got infected by SARS-CoV-2, since personal protection measures were strictly followed. In addition, his wife who took care of him during the hospitalization was not infected by SARS-CoV-2 either. The virus transmission chain of this patient was still unclear. One of the possibilities was that he might have been infected by asymptomatic carriers while receiving radiotherapy in another building of the hospital. To avoid transmission in the hospital, all patients visiting our hospital at present are required to wear face masks, permit measurement of their body temperature, and provide their “electronic health code” (a track log-based application) before entering the outpatient clinic. In addition, a chest CT and SARS-CoV2 nucleic acid test are required to be completed if they undergo hospitalization.

In the context of the current COVID-19 pandemic, therapies of countless cancer patients have been suspended and postponed due to medical service interruption or fear of contracting SARS-CoV-2 infection while seeking medical services. A flexible treatment plan should be developed based on balancing the risks and benefits according to the local epidemic situation and the patient's condition. In conclusion, this report describes the experience of a nasopharyngeal cancer patient with multiple comorbidities who underwent radiochemotherapy and still recovered from COVID-19, which may perhaps help soothe the excessive panic among patients and bring inspiration to doctors.

## Data Availability Statement

The raw data supporting the conclusions of this article will be made available by the authors, without undue reservation.

## Ethics Statement

Ethical review and approval was not required for the study on human participants in accordance with the local legislation and institutional requirements. The patients/participants provided their written informed consent to participate in this study. Written informed consent was obtained from the individual(s) for the publication of any potentially identifiable images or data included in this article.

## Author Contributions

NW and DW: study concept and design. WQ: data collection. FY and XR: data collection and analysis assistance. NW: drafting of the manuscript. DW and ZL: critical revision of the manuscript for important intellectual content. All authors: analysis or interpretation of data.

## Conflict of Interest

The authors declare that the research was conducted in the absence of any commercial or financial relationships that could be construed as a potential conflict of interest.

## References

[1] Liang W Guan W Chen R Wang W Li J Xu K. Cancer patients in SARS-CoV-2 infection: a nationwide analysis in China. Lancet Oncol. (2020) 21:335–7. 10.1016/S1470-2045(20)30096-6 32066541 PMC7159000

[2] Yu J Ouyang W Chua MLK Xie C SARS-CoV-2 transmission in patients with cancer at a tertiary care hospital in Wuhan, China. JAMA Oncol. (2020) 6:1108–10. 10.1101/2020.02.22.20025320 32211820 PMC7097836

[3] Zhang L Zhu F Xie L Wang C Wang J Chen R. Clinical characteristics of COVID-19-infected cancer patients: a retrospective case study in three hospitals within Wuhan, China. Ann Oncol. (2020) 7:894–901. 10.1016/j.annonc.2020.03.296 32224151 PMC7270947

[4] Wang W Xu Y Gao R Lu R Han K Wu G. Detection of SARS-CoV-2 in different types of clinical specimens. JAMA. (2020) 323:1843–44. 10.1001/jama.2020.3786 32159775 PMC7066521

[5] Yan CH Faraji F Prajapati DP Boone CE DeConde AS. Association of chemosensory dysfunction and COVID-19 in patients presenting with influenza-like symptoms. Int Forum Allergy Rhinol. (2020) 10:806–13. 10.1002/alr.22579 32279441 PMC7262089

[6] Menni C Sudre CH Steves CJ Ourselin S Spector TD. Quantifying additional COVID-19 symptoms will save lives. Lancet. (2020) 395:e107–8. 10.1016/S0140-6736(20)31281-2 32505221 PMC7272184

[7] Qin C Zhou L Hu Z Zhang S Yang S Tao Y Dysregulation of immune response in patients with COVID-19 in Wuhan, China. Clin Infect Dis. (2020) 71:762–8. 10.1093/cid/ciaa248 32161940 PMC7108125

[8] Sethuraman N Jeremiah SS Ryo A. Interpreting diagnostic tests for SARS-CoV-2. JAMA. (2020) 323:2249–51. 10.1001/jama.2020.8259 32374370

